# Fabrication of Graphene Nanomesh FET Terahertz Detector

**DOI:** 10.3390/mi12060641

**Published:** 2021-05-31

**Authors:** Yuan Zhai, Yi Xiang, Weiqing Yuan, Gang Chen, Jinliang Shi, Gaofeng Liang, Zhongquan Wen, Ying Wu

**Affiliations:** 1 Intelligent Technology and Engineering, Chongqing University of Science and Technology, Chongqing 401331, China; cquzhy@cqust.edu.cn (Y.Z.); xiangyicq@163.com (Y.X.); 13608335626@163.com (J.S.); 2 College of Optoelectronic Engineering, Chongqing University, Chongqing 400044, China; ywqwiki@gmail.com (W.Y.); gchen1@cqu.edu.cn (G.C.); lgf@cqu.edu.cn (G.L.); wenzq@cqu.edu.cn (Z.W.)

**Keywords:** graphene nanomesh, Si_3_N_4_ dielectric layer, FET, terahertz detector

## Abstract

High sensitivity detection of terahertz waves can be achieved with a graphene nanomesh as grating to improve the coupling efficiency of the incident terahertz waves and using a graphene nanostructure energy gap to enhance the excitation of plasmon. Herein, the fabrication process of the FET THz detector based on the rectangular GNM (r-GNM) is designed, and the THz detector is developed, including the CVD growth and the wet-process transfer of high quality monolayer graphene films, preparation of r-GNM by electron-beam lithography and oxygen plasma etching, and the fabrication of the gate electrodes on the Si_3_N_4_ dielectric layer. The problem that the conductive metal is easy to peel off during the fabrication process of the GNM THz device is mainly discussed. The photoelectric performance of the detector was tested at room temperature. The experimental results show that the sensitivity of the detector is 2.5 A/W (@ 3 THz) at room temperature.

## 1. Introduction

Graphene, a conjugated carbon sheet arranged in a 2D hexagonal lattice [1] and an important alternative to extend the validity of Moore’s law of electrons in semiconductors [2], has good transmission performance, a larger volume miniaturization space and a lower cost by virtue of ultrahigh electron mobility and ultrathin material thickness [3,4,5]. Due to the ultrathin planar structure of graphene, the performances of graphene-based field-effect transistor (GFET) devices are not obviously reduced when they shrink in size. The fabrication of the device is compatible with current CMOS technology, making GFET a highly competitive choice for high-performance, high-integration chips in the future [6,7,8].

At present, terahertz technology is widely used in many areas, such as defense, medical diagnosis, security monitoring, communication technology and space exploration. The development of terahertz technology has put forward higher requirements for terahertz detectors. However, due to the inherent limitations of electron velocity, the performance of traditional microwave electronic transistors decreases rapidly as the frequency approaches to the terahertz (THz) band (>0.1 THz). It is also difficult for the infrared optical devices to have good applications at frequencies below 20 THz [9]. The special position of THz in the electromagnetic spectrum (both electronic and optical devices are involved) poses a severe challenge to the modern solid-state devices [10]. With high carrier mobility, tunable electronic properties and unique photoelectric properties, graphene provides a new idea for the research of terahertz direct detectors. Terahertz detectors based on the GFET structure are developed and reported [6,7,11,12,13].

Although some methods can open the band gap of graphene, such as using graphene nanoribbons and double-layer graphene or applying stress to graphene, due to a series of challenges it has not been able to achieve mass production in the existing semiconductor process [11,12,13,14]. So far, no mature solid-state devices have been widely used in the terahertz band. This study aims to solve the problem encountered in the process of graphene transfer and lamination. In the paper, high quality graphene materials were prepared by chemical vapor deposition (CVD). The large-area and uniform graphene nanomesh structure was fabricated by electron-beam lithography (EBL). The fabrication process of the terahertz detector based on the graphene nanomesh was designed and the terahertz detector was developed.

## 2. Fabrication Process of Graphene Terahertz Detector

Based on the principles of the graphene FET terahertz detector, the pitch size and dimensions of the graphene nanomesh and structural parameters are decided and described in Table 1.

**Figure 1:**
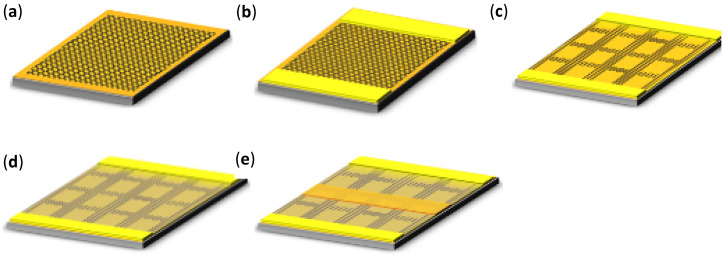
The fabrication process of the graphene terahertz field-effect transistor (FET) detector. (**a**) Chemical vapor deposition (CVD) preparation and substrate transfer of graphene. (**b**) Fabrication of source and drain electrodes. (**c**) Fabrication of graphene nanogrid by electron-beam lithography (EBL). (**d**) CVD deposition of the Si_3_N_4_ dielectric layer. (**e**) Fabrication of the gate electrode.

According to the device structure we designed, and the micro–nano processing platform of the National Center for Nanoscience and Technology of China, the overall fabrication process of the device is divided into the following five steps as shown in Figure 1.

### 2.1. Chemical Vapor Deposition (CVD) and Transfer of Graphene

Graphene film is prepared on copper foil (2 cm × 2 cm × 25 μm in volume) by CVD [4,15,16], and then transferred to the prepared Si/SiO_2_ substrate. Firstly, the desired copper foil substrate was obtained with the method of chemical immersion cleaning combined with electrochemical polishing. Then, graphene crystals were grown in a single-temperature-zone CVD tube furnace. The temperature was raised in a low-pressure environment (the pressure was set at 600–800 mTorr), and H_2_ was introduced when the temperature was raised. Under the condition of 1050 °C, the copper foil substrate was annealed for 1.5 h and maintained at this temperature, and H_2_ (catalyst) and CH_4_ (carbon source) were introduced for chemical reaction preparation. The reaction time was controlled within half an hour. After the graphene film was formed, the PMMA was coated as the protective layer. The thickness of the PMMA is about 200 nm when the spin speed is 4000 RPM and the concentration of the PMMA is 6%. Ferric chloride and hydrochloric acid were used as etching solutions to soak the copper foil substrate and some inorganic impurities. After copper foil etching, the PMMA/graphene was cleaned with deionized water. The selected substrate is a highly doped p-type silicon with a thickness of 525 μm. The thickness of the oxide layer above the silicon wafer is 285 nm. Before the transfer, the substrate was cleaned with deionized water and then dried with nitrogen. The PMMA/graphene was directly transferred to the substrate. At last, the PMMA was dissolved and removed in an acetone solution. In order to prevent graphene damage and avoid using ultrasound, the PMMA was removed by 80 °C water bath heating.

### 2.2. Fabrication of the Source and Drain Electrodes

Graphene film is essentially a single layer of carbon atoms. During the process design of the device, graphene damage should be avoided as far as possible. Therefore, the stripping process is chosen for electrode evaporation in order to avoid damage to the graphene caused by strong acid etching solutions in the electrode manufacturing process.

**Figure 2:**
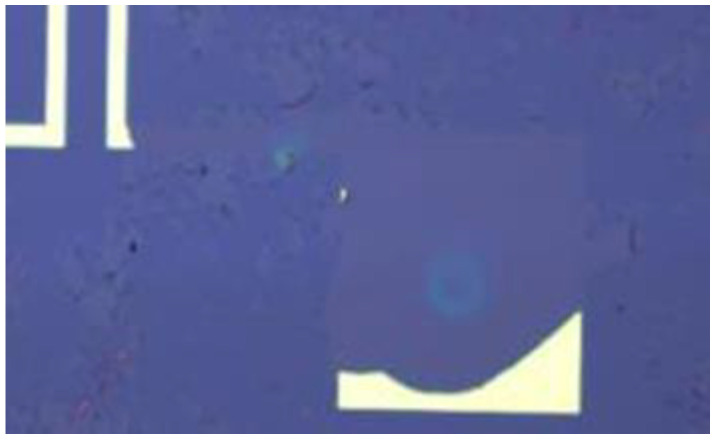
Metal shedding occurs when the graphene acts as a “stripping adhesive”.

However, the metal pattern formed on graphene by the stripping process is easy to fall off. The main reason is that the bonding between graphene and substrate is very low due to the van der Waals force. When the metal film is evaporated on graphene, the metal does not directly make contact with the substrate, and graphene may also act as a “stripping adhesive” in the stripping process to strip the metal directly. The phenomenon of metal peeling is observed obviously, especially in the location of the large-area metal pad as shown in Figure 2.

**Figure 3:**
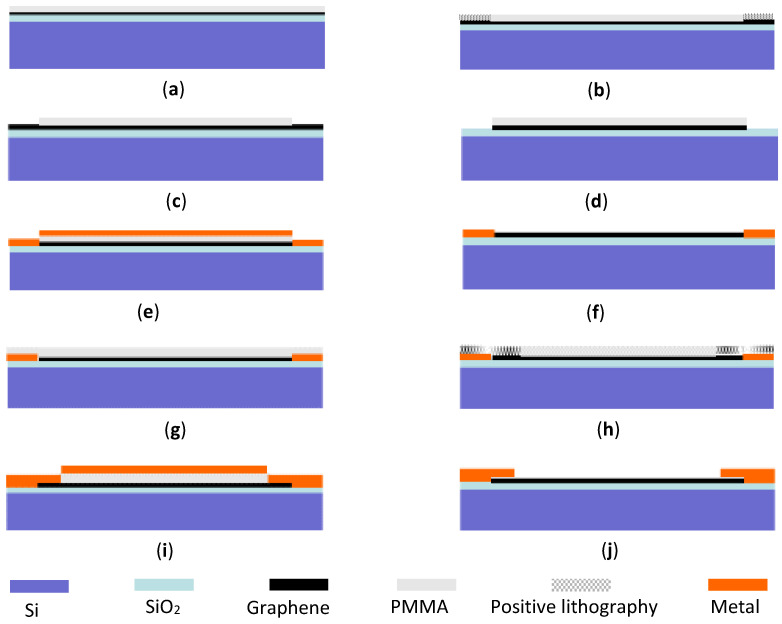
A schematic diagram of the source and drain electrode production process. (**a**) Spin-coating positive photoresist (**b**) Electron-beam lithography (**c**) Positive photoresist development (**d**) ICP etching (**e**) First evaporation of metal (**f**) Stripping (**g**) Spin-coating positive photoresist (**h**) Electron-beam lithography (**i**) Second evaporation of metal (**j**) Stripping.

In order to solve this problem, a process of etching graphene is added on the basis of the conventional stripping process, and then the metal evaporation stripping process is carried out twice. The optimized process is shown in Figure 3.

Before spin-coating, the graphene substrate is pretreated and soaked in acetone at room temperature for half an hour. After that, the graphene substrate is cleaned with isopropanol and deionized water, and dried with nitrogen successively. Next, the pre-baking temperature for the substrate is set to 120 °C and the heating time is 3 min. Then, a 4% concentration of PMMA (950 k) is selected as the positive glue to spin onto the substrate with a spin-coating speed of 5000 RPM. After spin-coating, the substrate is post-baked at 150 °C for 2 min, as shown in Figure 3a.

**Figure 4:**
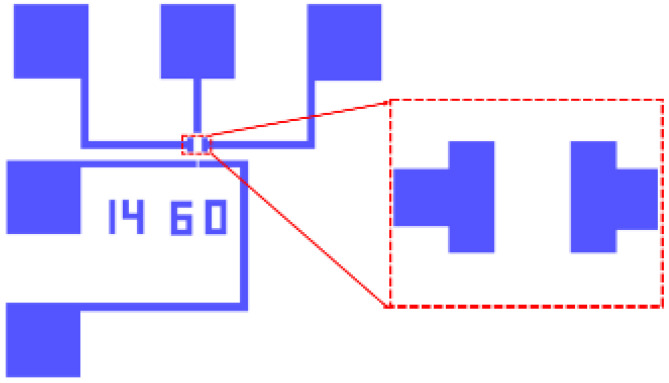
The layout of etching graphene under metal.

Figure 3b shows that the positive photoresist stripping process is used to expose the pattern of the metal wire and pad, in which the graphene would be removed, excluding the part of the source and drain electrodes located in the central part framed in the red dotted line. Figure 4 shows the layout pattern for electron-beam direct writing with an exposure dose of 850 μC/cm^2^. In order to improve the exposure efficiency, the electron-beam step is set at 50 nm.

Figure 3c shows the result of the positive photoresist development. The developer was a mixture of MIBK: IPA = 1:3. After 100 s of developing time, the substrate was then soaked in isopropanol for 50 s. In order to avoid the stripping effect of graphene upon the metal, oxygen plasma was used to etch the graphene with an etching time of 8 s and an etching power of 150 W under a pressure of 5 Pa. The development result is shown in Figure 3d. Then, the first evaporated metal of 8 nm/40 nm Ti/Au, shown in Figure 3e, could make contact with the SiO_2_/Si substrate to enhance the adhesion force. Meanwhile, the useless graphene easily fell off. Therefore, water bath heating and longer soaking time can be used to enhance the stripping effect without using ultrasound. The stripping result is shown in Figure 3f.

**Figure 5:**
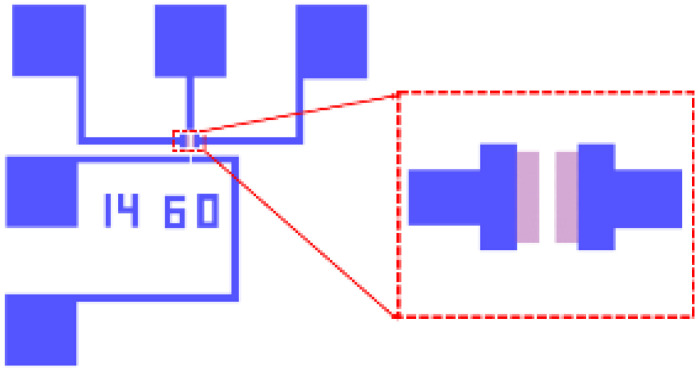
The layout of evaporating metal for the source and drain.

The same PMMA positive photoresist was used for spin-coating again. The peeling effect of the subsequent process could be enhanced by appropriately increasing the thickness of the spin-coating, as shown in Figure 3g. The source and drain electrodes are designed and shown in the enlarged picture in Figure 5. This scheme causes the metal to directly make contact with the substrate to enhance the adhesion of the metal, and it can also reduce the contact resistance by making the source and drain electrodes directly make contact with graphene, as shown in Figure 3h. The parameters of electron-beam exposure are the same as those of the first exposure.

The evaporated metal is Au with a thickness of 100 nm as shown in Figure 3i, including the patterns of wire and pad, and source and drain electrodes. In order to ensure the continuity of the whole metal pattern, the evaporation thickness is required to exceed the first overall thickness. Figure 3j shows the source and drain electrodes and metal pads obtained by the same stripping method presented above.

### 2.3. Fabrication of Graphene Nanomesh by EBL and OPE

**Figure 6:**
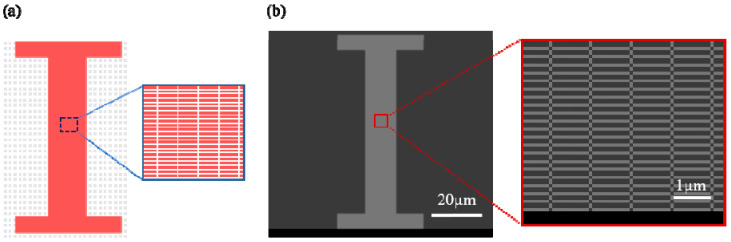
(**a**) Layout of rectangular graphene nanomesh (r-GNM) channel (**b**) SEM of rectangular graphene nanomesh (r-GNM) channel.

In order to obtain the large-area and uniform graphene nanogrid, the micro–nano fabrication method of electron-beam lithography (EBL) and oxygen plasma etching (OPE) was used to obtain the corresponding size of graphene nanogrid structure materials. Firstly, a large-area single-layer graphene was grown by chemical vapor deposition on a copper substrate. It was then transferred onto heavily doped p-type Si substrates with a 285 nm SiO2 layer using polymethyl methacrylate (PMMA)-assisted wet-transfer techniques. The silicon wafer (substrate size 1.2 cm × 1.2 cm) with the transferred graphene film was soaked in an acetone solution for 12 h, then soaked in isopropanol, slightly washed with deionized water, and finally dried (<80 °C). Then, the PMMA with a concentration of 4% was used as a resist, and the spin-coating speed was set at 5000–6000 RPM (the corresponding coating thickness was about 200 nm). For different structures and sizes of GNM, the corresponding exposure dose was determined and selected for electron-beam exposure. At last, a graphene nanomesh structure was fabricated by oxygen plasma etching. The layout of the graphene channel between the source and drain electrodes is shown in Figure 6a, which was processed by EBL [17]. The exploration of the graphene nanomesh is shown in the right image of Figure 6b. After the electron-beam direct writing exposure process is completed, the channel image of the device is obtained and shown on the left of Figure 6b.

**Figure 7:**
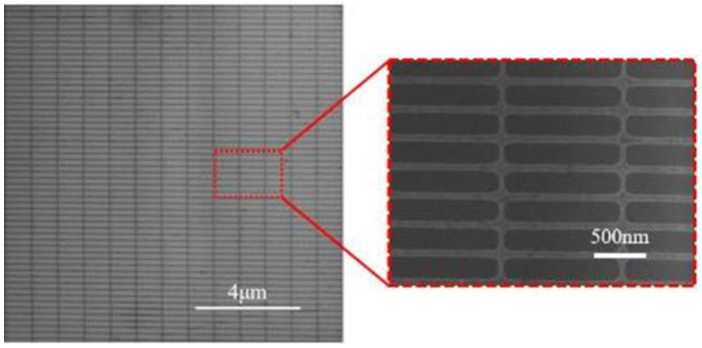
SEM images of the graphene nanomesh structure after oxygen plasma etching.

SEM images of the graphene nanomesh structure after oxygen plasma etching are shown in Figure 7. It can be seen that the fabricated graphene nanogrid can maintain the integrity of the periodic structure in the large-area.

### 2.4. CVD Deposition of the Dielectric Layer

As the device will work in THz frequency bands, the dielectric constant of the gate dielectric layer under the gate electrode should be as large as possible. For graphene that has been processed into nanostructure, the bonding ability with the dielectric layer on the surface of graphene and the question of easy damage should be considered carefully in the subsequent processes.

Therefore, plasma-enhanced chemical vapor deposition (PECVD) was chosen to deposit Si_3_N_4_ on the surface of the graphene nanomesh as the dielectric layer. Because the combination of Si_3_N_4_ and graphene is much better than that of a silicon oxide dielectric layer created by the thermal oxidation process, the dielectric constant of Si_3_N_4_ (ε = 6.6) is much higher than that of silicon oxide (ε = 3.9), and Si_3_N_4_ has a higher polarized photo–phonon frequency, which can reduce the phonon scattering of the graphene conductive channel and is conducive to the photoelectric application of graphene [18]. Simultaneously, the PECVD process worked at a low-temperature environment, which could effectively prevent the damage of graphene. The gas source of Si_3_N_4_ CVD was the inert gas of N_2_ and NH_3_. In addition, the power source was a low-density plasma with a power of only 40 W. These factors could ensure that the damage to graphene is minimized in the manufacturing process of the dielectric layer.

**Figure 8:**
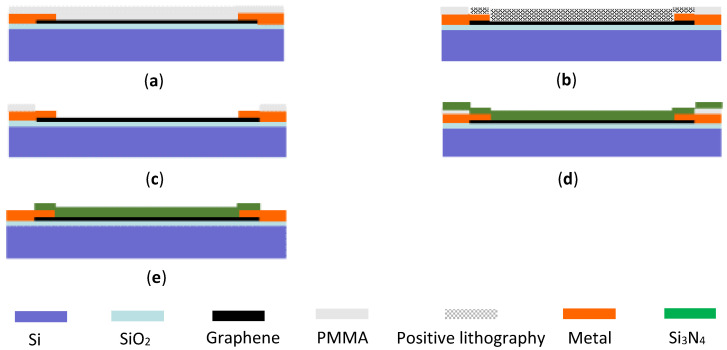
The preparation process of the dielectric layer. (**a**) Spin-coating positive photoresist (**b**) Electron-beam lithography (**c**) Positive photoresist development (**d**) CVD Si3N4 (**e**) Stripping.

The growth process of the Si_3_N_4_ dielectric layer is shown in Figure 8. The result of spin-coating positive photoresist is shown in Figure 8a. The rotation speed was set at 4000–5000 RPM to spin-coat with the PMMA 950 k positive photoresist and control the photoresist thickness above 200 nm. The pre-baking and post-baking time are 1–2 and 2 min, respectively. The pre-baking and post-baking temperatures are 120 and 150 °C, respectively. As the pattern of the dielectric layer is simple and the requirement of dimensional accuracy is low, only accurate alignment is required for electron-beam direct writing. The exposure dose of electron beam was 850 μC/cm^2^, and the step setting was 50 nm. The fabrication of EBL is shown in Figure 8b. The mixture of MIBK and IPA (1:3) was used as developer. The positive photoresist development time was 100 s, and isopropanol was used for fixing for 30 s. After the development was obtained, as shown in Figure 8c, the Si_3_N_4_ was grown by using the Si 500 D PECVD equipment (SENTECH). SiH_4_/N_2_ and NH_3_ were used as the gas source, and the reaction equation is:

3SiH_4_ + 4NH_3_ → Si_3_N_4_ + 12H_2_↑

While the RF power, temperature, pressure and deposition time are set to 40 W, 55 °C, 50 Pa and 1 h, respectively, the thickness of the Si_3_N_4_ film would be about 60 nm, as shown in Figure 8d. The last step of the dielectric layer deposition is stripping, which used an acetone solution to remove the PMMA, and the result is shown in Figure 8e.

### 2.5. Fabrication of the Gate Electrode

**Figure 9:**
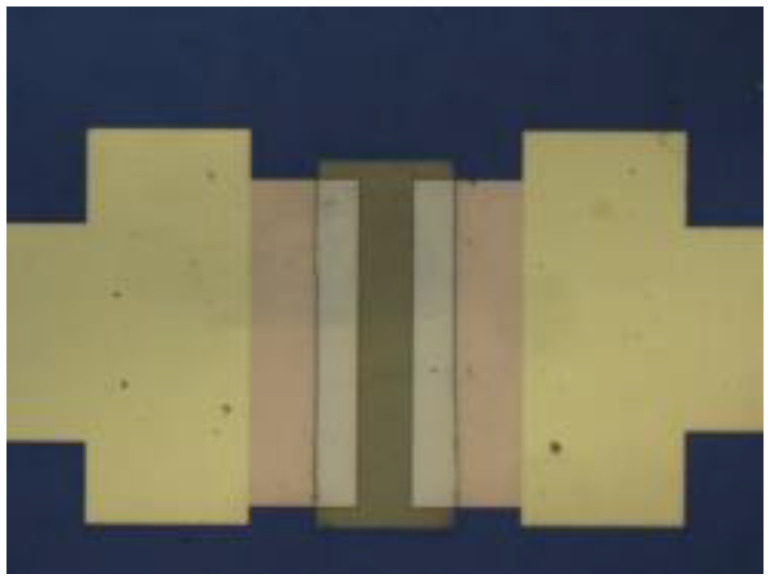
An optical photograph of the device after the fabrication of the dielectric layer.

**Figure 10:**
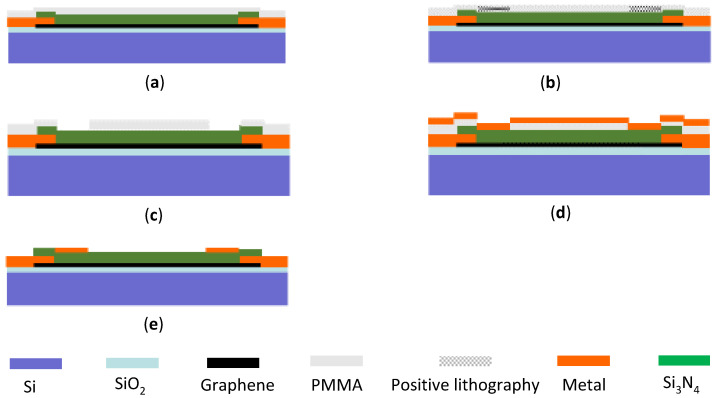
The process flow of the gate electrode. (**a**) Spin-coating positive photoresist (**b**) Electron-beam lithography (**c**) Positive photoresist development (**d**) Evaporating metal (**e**) Stripping.

After the fabrication of the dielectric layer Si_3_N_4_, the graphene nanomesh was covered by silicon nitride, and the possibility of damage was reduced, as shown in Figure 9. Therefore, the conventional metal evaporation stripping process can be used for the fabrication of different gate electrodes. The specific process flow is shown in Figure 10.

Figure 10a shows the spin-coating result, among which PMMA (950 k) stock solution was used for spin-coating, the spin-coating speed was 4500 RPM, and the thickness of the adhesive was controlled above 200 nm. The pre-baking and post-baking time both were 100 s, and the temperatures were set to 80 and 150 °C, respectively. While the electron-beam exposure dose was set at 900 μC/cm^2^ and the step was set at 50 nm, the overlay precision could be ensured for the electron-beam direct writing exposure of the gate electrode pattern. The EBL result is shown in Figure 10b. The positive development still used the mixture of MIBK and IPA (1:3) as developer in Figure 10c. The development time was 100 s and then isopropanol was used for fixing. The thickness of the evaporated gate metal should be as thin as possible, as shown in Figure 10d. Therefore, we evaporated 10/50 nm thick Ti/Au metal, which is conducive to complete stripping. The PMMA adhesive was removed by immersing the substrate in an acetone solution to complete the stripping process, as shown in Figure 10e. A water bath with long-time soaking was used to ensure complete stripping, because due to the narrow metal spacing it was easy to cause incomplete stripping.

**Figure 11:**
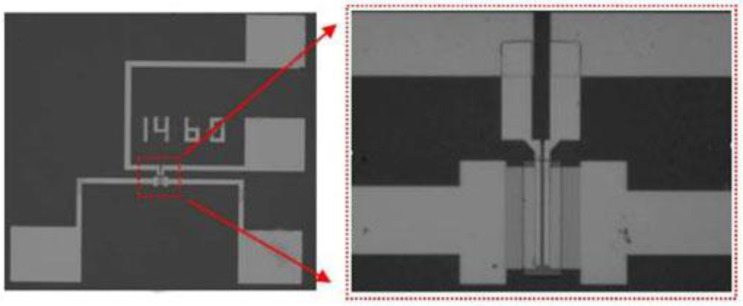
Optical photographs of the top gate FET device.

Using the above process, we fabricated an r-GNM FET THz device with a graphene nanomesh size of 14 nm × 60 nm, which is shown in Figure 11.

## 3. Measurement Experiments

**Figure 12:**
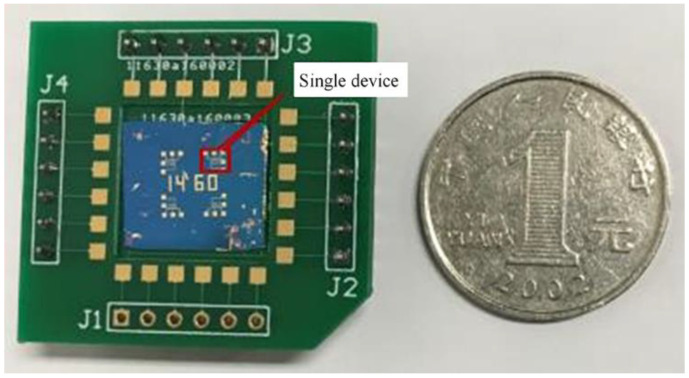
Physical drawings of devices on PCB.

According to the size of the silicon substrate, the PCB board is designed for the convenience of testing the electrical properties and terahertz detection of the FET device. Figure 12 shows that the metal pads of the device are connected to the pins of the PCB board by gold wires.

### 3.1. Electrical Transfer Characteristics

**Figure 13:**
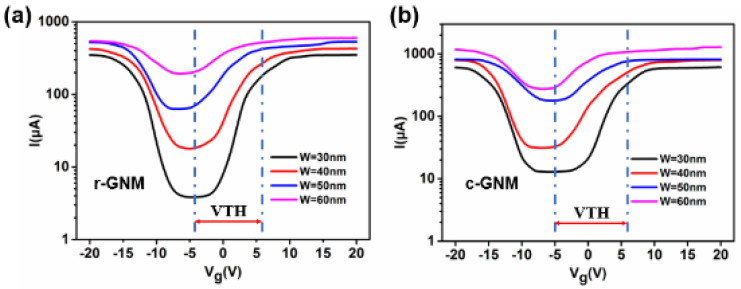
GNM transfer characteristic curves with dimensions of 30, 40, 50 and 60 nm, respectively. (**a**) r-GNM (**b**) c-GNM.

The transfer characteristics of the Gr layer are shown in Figure 13. Figure 13a shows the transfer characteristics at Vds = 2 V for the devices based on r-GNMs with different neck widths of 30, 40, 50, and 60 nm, from which we could determine the corresponding Ion/Ioff ratios of ~40, ~25, ~5, and ~4, respectively. The transfer characteristics for the devices based on c-GNMs with different neck widths of 30, 40, 50, and 60 nm are presented in Figure 13b. Comparing Figure 13a with Figure 13b, we can see that the conduction current of c-GNMs is much larger than that of r-GNMs (about two times). As a result of GNM being able to be viewed as an interconnected network structure of graphene, the actual area of c-GNM delivering current is greater than that of r-GNM, which leads to the current of c-GNM being greater than r-GNM under the same conditions. Additionally, the Ion/Ioff ratios of r-GNMs with different neck widths of 30, 40, 50, and 60 nm obtained were ~100, ~25, ~8, and ~3, respectively, indicating that the Ion/Ioff ratio of the GNM-based devices can be readily tuned by varying the neck width, which plays an important role in charge of transport properties.

### 3.2. Terahertz Photocurrent Measurement

**Figure 14:**
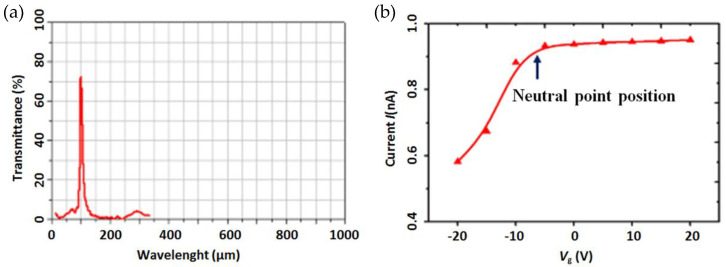
(**a**)The terahertz transmission of BPF3.0 (**b**) the curves of the photocurrent of the FET terahertz detector versus the gate voltage.

The IR-30 steady-state infrared light source (HawkEye Technologies) was used as the light source for the THz response test. The excitation source of the light source was powered by 2.5 V DC and could be regarded as a blackbody light source at a stable working state. A 3 THz band-pass filter is used behind the light source to obtain a 3 THz electromagnetic radiation wave. The BPF3.0 filter (TYDEX) was used to obtain the transmission spectrum, which is shown in Figure 14a. In order to obtain the influence of the gate voltage on the terahertz photocurrent, the gate voltage was changed to test the photocurrent for r-GNM with a 60 nm width. The gate voltage had a decisive effect on the dispersion of the graphene terahertz plasmon by changing the Fermi level, but the effect on the terahertz absorption of the graphene nanomesh was not obvious.

Figure 14b shows that the increase in gate voltage increased the photocurrent, but the increase after the neutral point tends to be saturated. The reason is that the increase in gate voltage will also increase the Fermi energy level, and then affect the carrier concentration, so that the excitation of graphene plasmon is weak when the Fermi energy level is relatively low, resulting in a slightly small photocurrent. When the Fermi level increases to a certain extent, the saturation of the terahertz absorption makes the photocurrent no longer increase.

In order to further determine the response rate of the graphene nanomesh FET terahertz detector, the standard Golay cell GC-1P terahertz detector (TYDEX) was used to calibrate the power of the terahertz radiation source by replacing the graphene nanomesh FET terahertz detector in the same position. According to the voltage sensitivity formula:

(1) $$R_{V}=\frac{V}{P_{o}}$$

where *R_V_* is the hardness of high Leghorn. When the reference frequency was 10 Hz, the photovoltage signal intensity measured by the Golay GC-1P was 6.47 μW. Under this condition, the sensitivity of the Golay was 81.3 KV/W, and the output optical power *P_o_* was about 0.08 μW. The current sensitivity formula of the detector is as follows:

(2) $$R_{i}=\frac{i}{P_{in}}$$

where *R_i_* is the current sensitivity of the detector, *i* is the detection current, and *P_in_* is the actual incident light power. The current sensitivity of the graphene terahertz FET detector is 12 mA/W under 3 THz radiation.

## 4. Conclusions

Monolayer graphene was grown on copper foil by CVD, and the high-quality graphene film was obtained by wet transfer on the SiO_2_/Si substrate. Then, aiming at the problems of electrode stripping, the method of metal evaporation and photoresist stripping twice was used to realize the source and drain electrode lamination of graphene, and enhance the adhesion of the metal wire and electrodes with the substance. Next, the graphene nanomesh with a large-area and uniform size was fabricated by EBL and oxygen plasma etching. Lastly, the conventional metal evaporation stripping process was used to fabricate the gate electrode on the Si_3_N_4_ dielectric layer. In sum, the fabrication process of the terahertz detector based on the graphene nanomesh was designed and the terahertz detector was developed. The electrical characteristics of the graphene nanomesh were tested, which verified the regulating effect of the graphene nanomesh on the electronic energy gap. A test system was built to test the developed detector, suggesting that the THz detection sensitivity of the detector can reach 2.5 A/W at room temperature.

## Figures and Tables

**Table 1:** Parameters of the graphene FET terahertz detector.

Parameters of Graphene	Value
Minimum width of nanostructures	30 nm~60 nm
Period of structural array	1 μm
Channel length	14 μm
Channel width	60 μm
Thickness of dielectric layer	60 nm
